# Medical students' perspective on the United States Medical Licensing Examination (USMLE) Step 1 transition to Pass/Fail

**DOI:** 10.12688/mep.19975.2

**Published:** 2024-07-02

**Authors:** Samiksha Prasad, Christine Perez, Kate J.F. Carnevale

**Affiliations:** 1 Dr. Kiran C. Patel College of Allopathic Medicine, Nova Southeastern University, Fort Lauderdale, Florida, 33314, USA

**Keywords:** pass/fail, wellness, medical student, study approach, pre-clerkship curriculum, standardized examination, board certification

## Abstract

**Background:**

The transition of the United States Medical Licensing Exam: Step 1 to Pass/Fail (P/F), from scored, caused uncertainty about students’ preparedness and wellbeing related to the exam. Comparison of study behavior and results, before and after the P/F transition can provide insights for the medical curriculum and student support. The aim of this study is to evaluate students’ perceptions of their efforts in Step 1 preparation, as compared to cohort performances to determine the impact of the transition of Step 1 to P/F on medical students.

**Methods:**

Data from four cohorts of second-year medical students (Class of 2022–25, N = 204) were collected from their dedicated Step 1 self-study block. Student study regiments, aggregate practice test results, Step 1 pass rates and post-block self-reported surveys were analyzed qualitatively to compare student effort and outcomes for scores and P/F cohorts.

**Results:**

Analysis of practice exam averages across the four student cohorts demonstrates a potentially slower and less rigorous start to Step 1 self-studying during the dedicated preparation block for the cohorts that took the P/F Step 1 exam format as compared to the previous cohorts that prepared for a scored Step 1 exam. Similarly, self-reported study regiments decreased in the median number of hours/day and number of weeks of study for the cohorts with P/F Step 1 exam. There was also a slight shift in the type of study resources used, between the two groups, with the scored group using more traditional board preparation resources.

**Conclusions:**

The P/F transition of the Step 1 exam may lead to reduced student preparedness and may require adjustments in the resources and support provided by institutions.

## Introduction

In the United States, medical students hoping to become practicing physicians must demonstrate competency in medical knowledge (MK), critical thinking skills (CTS) and patient care (PC) by successfully completing several standardized examinations throughout their medical training. The United State Medical Licensing Examination (USMLE) is a 3-part examination designed to assess MK of the medical school students and the application of the MK to PC though multiple-choice questions. The 3 parts consist of Step 1, Step 2 clinical knowledge (CK) and Step 3. Step 1 is typically taken at the end of the second year of medical school and is followed by the Step 2 CK at the end of the third year (or may also be taken at some point during the fourth year) of medical school training. Step 3 is taken after the successful completion of Step 1 and Step 2 CK, once students have completed their MD or DO degree, and typically near the end of the 1st year of GME residency training. This information is available on the USMLE website.

The high stakes nature of the USMLE has been reported to drive medical student stress and mental or emotional burnout, as well as fueling toxic competition among medical students during their training ( Chen *et al.*, 2019). Until recently, Step 1 has been arguably one of the most pivotal exams a future physician must conquer during medical school. Despite the initial design of the USMLE to assist state authorities in granting medical licenses, the emphasis of these assessments has evolved beyond the intended pass/fail indication of competence ( Prober *et al.*, 2016). The Step 1 three-digit scores had become a heavily influential metric for residency and clinical research directors as well as other opportunity granting bodies for medical students and recent graduates. This single exam outcome appeared to be unintentionally imbued with the power to make or break career plans of aspiring physicians at a very early point in their medical training ( McGaghie *et al.*, 2011). Considering this and other factors, the decision was made by the National Board of Medical Examiners (NBME) to transition the USMLE Step 1 to a non-scored exam. As of January 26th, 2022, Step 1 exam scoring format officially became Pass/Fail (P/F) and will no longer be able to function as the primary determinant of a medical student’s competitiveness for residency applications and other meritorious awards.

While this decision was mostly celebrated by medical school students, faculty and administrators, concerns exist about potential unintended consequences of the USMLE Step 1 paradigm shift to P/F ( Girard *et al.*, 2022). One such consequence may be disadvantageous changes in student study approaches, that might impact students’ overall medical competency, in favor of engaging in activities with the potential to help student appear more competitive by other metrics used for ranking in residency programs ( Carmody *et al.*, 2021). According to recent literature, most students report that they anticipate spending less time studying and more time needing to diversify their residency application portfolios ( Cangialosi *et al.*, 2021; Girard *et al.*, 2022; Mott *et al.*, 2021). These changes may more drastically reshape the nascent academic cultures of new medical schools whose student bodies lack generations of vetted alumni with track records beyond residency that can inspire confidence and promote stability of study practices within current medical school undergraduates. As this change has acutely affected the most recent class of medical students, it is timely and necessary to understand the complex outcome of the transition to P/F and how it may affect the academics, prospects, and wellbeing of medical students, especially at a new medical school. The aim of this study is to evaluate students’ perceptions of their efforts in Step 1 preparation, as compared to cohort performances on practice and Step 1 exams, to determine the impact of the transition of Step 1 to P/F on medical students.

## Methods

### Study design and setting

To evaluate potential change in study strategies for second year medical students between cohorts who were assessed by the scored versus pass/fail formats of the USMLE Step 1 board examinations, analyses of cohort average practice scores, USMLE Step 1 pass rates, and self-reported evaluations of time spent studying and resources used were conducted..

All the participants of the study were enrolled in a mandatory self-study dedicated Step 1 exam preparation course which is part of the medical school curriculum. As part of the study block, students were required by the curriculum to develop a highly organized study plan including intended weeks of study and hours per day of study. This study plan was reviewed and approved by the administration (Director of Student Success) ahead of the dedicated study block. During their pre-clerkship education, the students were also provided with several opportunities to take official practice tests through the National Board of Medical Examiners (NBME) i.e. Comprehensive Basic Science Examination (CBSE), USMLE Comprehensive Basic Science Self-Assessment (CBSSA), and third-party USMLE World (UWorld®), along with other provided resources. These exam scores were used to map the overall cohort trajectory and progress throughout their preparation periods.

### Sample participants

Second year, second semester medical students in the Dr. Kiran C. Patel College of Allopathic Medicine at Nova Southeastern University in Fort Lauderdale, FL USA were enrolled in a dedicated USMLE Step 1 study block. Students were compared from the Class of (C/O) 2022 through C/O 2025. Average entrance Medical College Admissions Test (MCAT) scores had a mean of 511±1 and undergraduate college Grade Point Averages (GPA) had a mean of 3.65±0.5, for each cohort, demonstrating comparable student pre-matriculation performance and abilities. Students in their second year of undergraduate medical education used practice tests to quantitatively evaluate their study progress and potential trajectory for success on the USMLE Step 1 exam. The students were then able to make self-corrections based on the available feedback. The USMLE CBSE 1 and 2 practice exams were taken in the summer before and fall of their second year in medical school. The USMLE CBSSA 1 and 2 and UWorld® 1 & 2 practice exams were staggered between January and March of their second year in medical school. Students’ privacy was ensured by anonymous data collection of course evaluation data and aggregate practice and Step 1 exam data, along with cohort pass rates, in accordance with the study protocol that was confirmed to be exempt by the NSU IRB (NSU- IRB # 2022-119).

### Data collection

Cohort pass rates for Step 1, a 14-item anonymous final course evaluation form (available at Harvard Dataverse) (including self-reported resources used, hours per day and number of weeks spent studying), and cohort practice test data were analyzed for comparative analysis to determine if there were any trends and correlations before and after the transition to Pass/Fail (P/F) for Step 1. Practice tests that were given were the USMLE Comprehensive Basic Science Examination (CBSE), USMLE Comprehensive Basic Science Self-Assessment (CBSSA), UWorld® question bank practice tests, each with a pair of practice exams given at staggered timepoints in the students' undergraduate medical education. Both CBSE practice tests were taken prior to the Step 1 study block, which began in January of the second year. Both CBSSA and UWorld practice tests were taken between January and March of the second year. Access to the data instrument is available at Harvard Dataverse: Step 1 Preparation Student Evaluation Questionnaire, https://doi.org/10.7910/DVN/LOT6TD ( Carnevale, 2024).

### Data analysis

Cohort averages for practice test scores and overall pass rates for Step 1 between cohorts were compared. Anonymous student evaluations were analyzed for self-reported time per day and weeks spent studying during the dedicated Step 1 preparation block. These values were compared to cohort averages and cohort test results. Student evaluations were also qualitatively analyzed for self-reported study resources used, to see if trends changed from year to year, especially before and after the transition of Step 1 to P/F. All data was analyzed using Microsoft Excel 365 (version 16.71). Access to the data used in the study is available at Harvard Dataverse: Step 1 Prep Data Prasad Perez Carnevale. https://doi.org/10.7910/DVN/IXIUMB ( Carnevale, 2023).

### Human subjects

The Nova Southeastern University Internal Review Board granted exempt status and waived individual consent for this study (NSU- IRB # 2022-119). Student participant privacy was protected by only obtaining deidentified data and cohort averages for this study.

## Results

**Figure 1.**
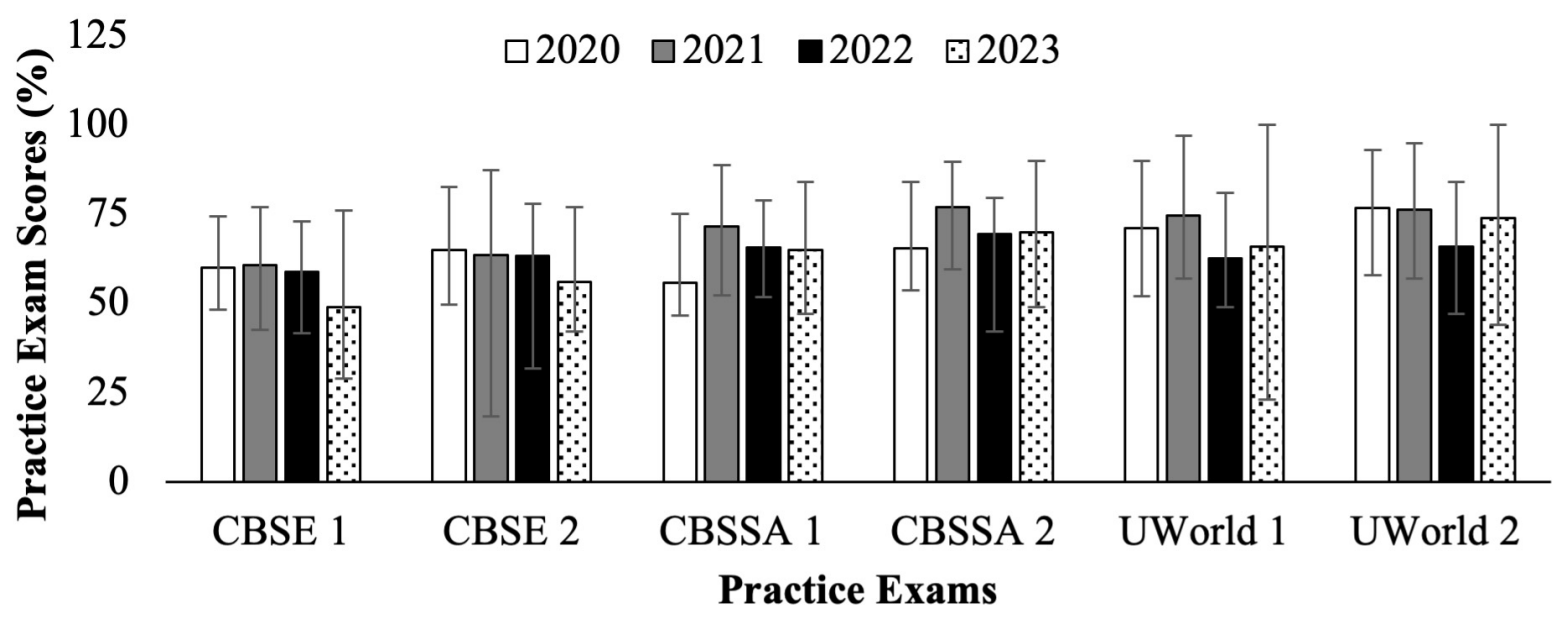
Average practice exam scores (%) for students who took Step 1 in 2020 – 2023. Showing National Board of Medical Examiners (NBME) Comprehensive Basic Science Examination (CBSE), NBME Comprehensive Basic Science Self-Assessment (CBSSA), and UWorld practice tests. Error bars depict high and low scores for each exam cohort. Cohorts in 2020 (white) & 2021 (gray) took the scored Step 1, and in 2022 (black) & 2023 (pattern) took the Pass/Fail (P/F) Step 1. Range bars represent high and low scores for each cohort. *Note: the Class of (C/O) 2025 CBSE and CBSSA exam scores were reported as weighted averages rather than direct percentages.*

Students in their second year of undergraduate medical education used practice tests to quantitatively evaluate their study progress, and potential trajectory for success on the USMLE Step 1 exam. The students were then able to make self-corrections based on the available feedback. The USMLE CBSE 1 and 2 practice exams were taken in the summer before and fall of their second year in medical school. The USMLE CBSSA 1 and 2 and UWorld® 1 & 2 practice exams were staggered between January and March of their second year in medical school. Figure 1 ( Carnevale, 2023) displays the cohort averages for each practice test for the C/O 2022 through 2025. The C/O 2022 and 2023 took their USMLE Step 1 as scored in exams in 2020 and 2021 (white and gray bars in Figure 1), respectively. The C/O 2024 and 2025 took their USMLE Step 1 as P/F in exams in 2022 and 2023 (black and pattern filled bars in Figure 1), respectively. No statistically significant difference was observed in cohort performance averages before and after the transition to P/F. However, it can be seen that the distributions between high and low scores were wider for the cohorts preparing for the P/F Step 1 (black and pattern fill bars, Figure 1) than for cohorts who prepared for the scored Step 1 exam (white and grey fill bars, Figure 1). Of note, the C/O 2025 CBSE and CBSSA exam scores were reported as weighted averages rather than direct percentages, so differences can only be inferred rather than directly compared, since the cohort score values provided were not on the same scale of raw percentage.

**Figure 2.**
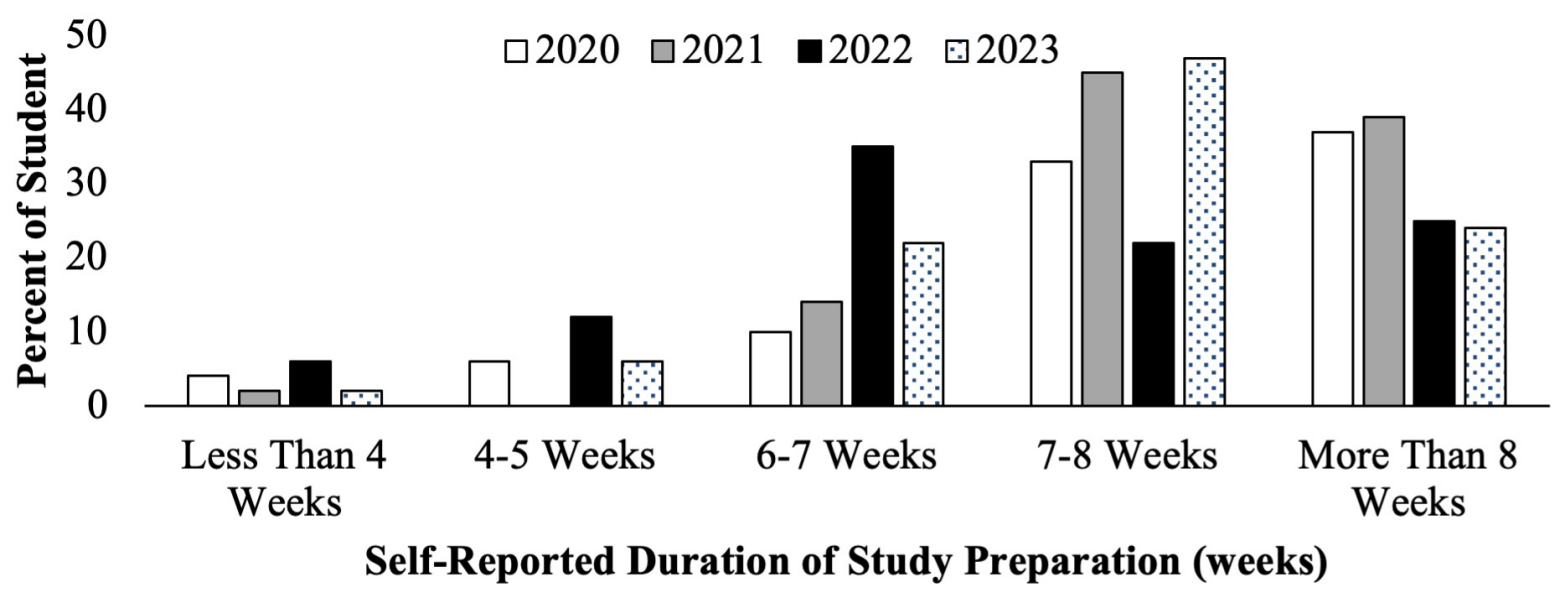
Course evaluation results of student self-reported study schedule weeks of study during the Step 1 dedicated study block by year: cohorts in 2020 (white) & 2021(grey) took the scored Step 1, and in 2022 (black) & 2023 (pattern) took the Pass/Fail (P/F) Step 1.

 The second-year medical students filled out course evaluations at the conclusion of their Dedicated Step 1 Study Block course. Student self-reported number of weeks spent in Step 1 study during the Step 1 dedicated study block are seen below ( Figure 2). Cohorts who prepared for the scored Step 1 exam in 2020 and 2021 ( Figure 2, white and gray bars) had a higher proportion of students reporting studying for greater than 7–8 weeks compared to students who prepared for P/F USMLE Step 1 in 2022 and 2023 ( Figure 2, black and pattern bars) who reported studying for fewer weeks, on average. Since the data obtained was deidentified, there was no way to correlate individual student effort and exam performance outcomes, but it may be inferred that more weeks of studying would likely lead to a higher level of preparedness, compared to fewer weeks of studying.

**Figure 3.**
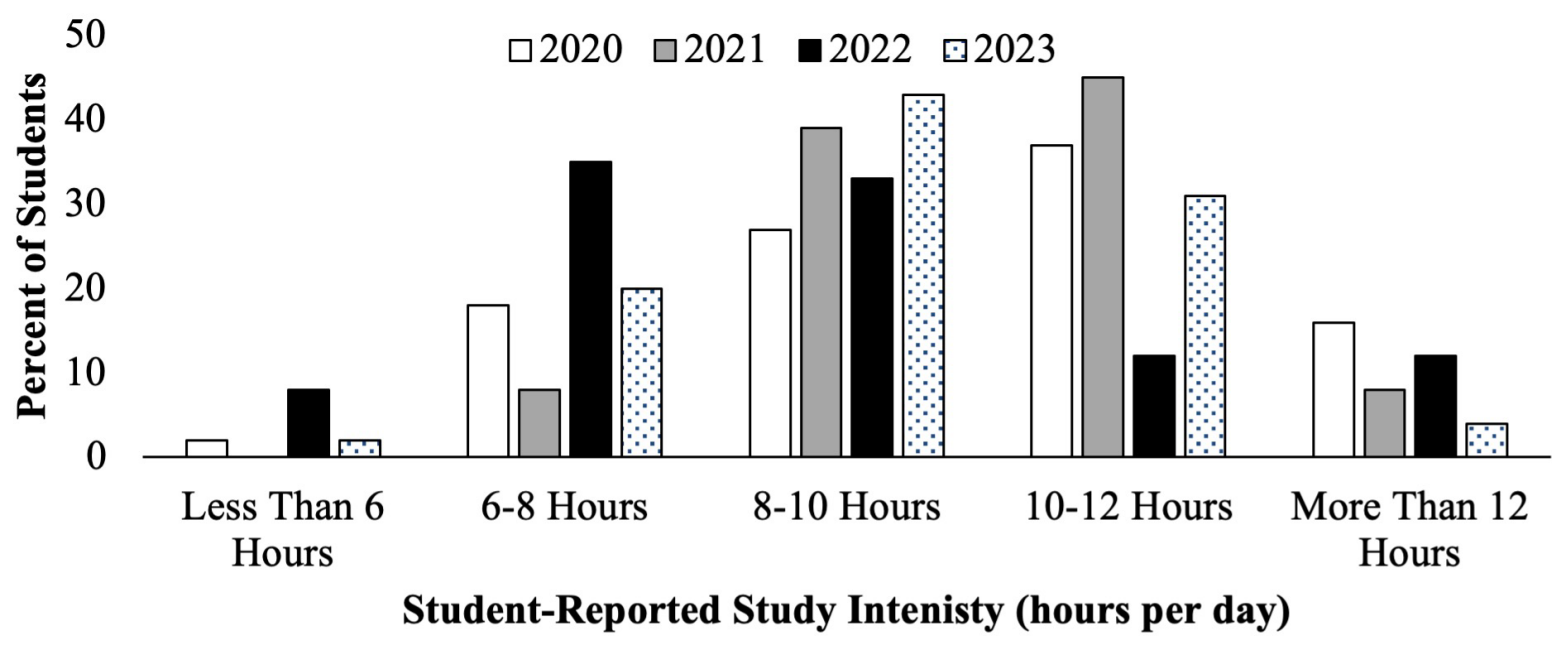
Course evaluation results of student self-reported study schedule hours of study per day during the Step 1 dedicated study block by year: cohorts in 2020 (white) & 2021(grey) took the scored Step 1, and in 2022 (black) & 2023 (pattern) took the Pass/Fail (P/F) Step 1.

Similarly, the second-year medical students course evaluations showed responses concerning self-reported hours per day spent studying, seen below ( Figure 3). Cohorts who prepared for the scored Step 1 exam in 2020 and 2021 ( Figure 3, white and gray bars) had a higher proportion of students reporting studying for greater than 10–12 hours per day compared to students who prepared for P/F USMLE Step 1 in 2022 and 2023 ( Figure 3, black and pattern filled bars), of whom the majority reported studying for 6–10 hours per day. Since the data obtained was deidentified, there was no way to correlate individual student effort and exam performance outcomes, but it may be inferred that more prolonged daily studying could be a wellness concern, as less time would then be available for fitness and social wellbeing activities.

**Figure 4.**
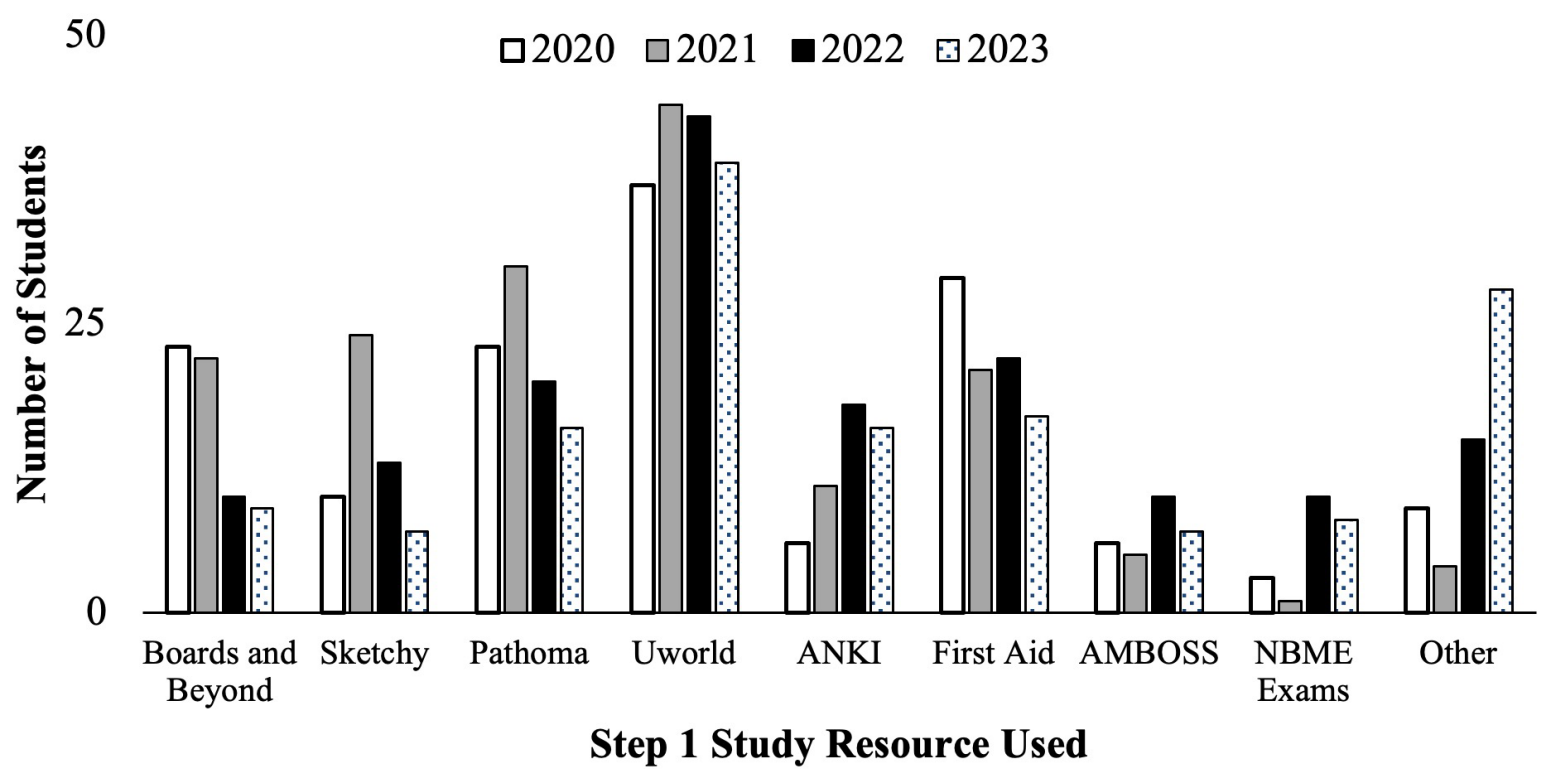
Student self-reported study resources used (number of students) during the Step 1 dedicated study block by year: cohorts in 2020 (white) & 2021(grey) took the scored Step 1, and in 2022 (black) & 2023 (pattern filled) took the Pass/Fail (P/F) Step 1. *Other includes the following resources: High Yield, Reddit, Pixorize, Physeo, Notes, Dirty Medicine (You Tube), Dr. Neil (YouTube), Osmosis, Costanzo, Textbooks, Becker, Lightyear, Kaplan, Med school Bootcamp, HyGuru, Podcast.

The Step 1 Dedicated Study Block course evaluations were also used to query second-year medical students about the types of study resources they used to prepare for the USMLE Step 1. Student self-reported resources used during their study block are seen below ( Figure 4 & Table 1). All cohorts reported they most utilized the UWorld® question bank to study for Step 1. However, cohorts who prepared for the scored Step 1 exam in 2020 and 2021 ( Figure 4, white and gray bars) on average had a higher proportion of students reporting studying using Pathoma, Boards and Beyond, First Aid and Sketchy, compared to students who prepared for P/F USMLE Step 1 in 2022 and 2023 ( Figure 4, black and pattern filled bars) for whom greater proportions reported studying with ANKI, Amboss, and Other* resources (i.e., High Yield, Reddit, Pixorize, Physeo, Notes, Dirty Medicine (YouTube), Dr. Neil (YouTube), Osmosis, Costanzo, Textbooks, Becker, Lightyear, Kaplan, Med school Bootcamp, HyGuru, Podcast), compared to the previous cohorts. As far as the authors are aware, all listed resources were available to all cohorts of students.

**Table 1.** Student self-reported study resources used during dedicated United State Medical Licensing Examination (USMLE) Step 1 self-study block for the Class of (C/O) 2022 and 2023, who took the scored Step 1, and the C/O 2024 and 2025, who took the Pass/Fail (P/F) Step 1. *Other includes the following resources: USMLERx, High Yield, Reddit, Pixorize, Physeo, Notes, Dirty Medicine (You Tube), Dr. Neil (YouTube), Osmosis, Costanzo, Textbooks, Becker, Lightyear, Kaplan, Med school Bootcamp, HyGuru, Podcast which were used by less than 5% of the groups, each.

Study Resource	Scored Exam Takers (%)	Pass/Fail Exam Takers (%)
**UWorld**	26.3	26.6
**First Aid**	16.2	12.7
**Pathoma**	17.2	11.7
**ANKI**	5.5	11.0
**Sketchy**	11.0	6.5
**Boards and Beyond**	14.6	6.2
**NBME Exams**	1.3	5.8
**AMBOSS**	3.6	5.5
Other *	4.3	14

As seen in Table 1, the top five most used resources for the C/O 2022 & 2023 were UWorld (26.3%), Pathoma (17.2%), First Aid (16.2%), Boards and Beyond (14.6%) and Sketchy (11.0%). This is compared to the top five resources for the C/O 2024 & 2025 being UWorld (26.6%), First Aid (12.7%), Pathoma (11.7%), ANKI (14.6%), and Sketchy (6.5%), with the combination of Other* resources amounting to 14.0% ( Table 1). Students who prepared for the scored Step 1 exam utilized more traditional board preparation resources, such as Boards and Beyond® (14 % Scored vs 5.8 % P/F), Pathoma® (16.5 % Scored vs 11 % P/F) and First Aid ® (15.6% Scored vs 12 % P/F) compared to students who took the P/F Step 1 exam ( Table 1). Results indicated that for P/F exam format preparation, students utilized more diverse resources (such as MedSchool Bootcamp, Physio® and HyGuru®) as well as more practice exam resources. Despite the transition to P/F, some study resources have maintained their value to students, such as the UWorld question bank.

**Figure 5.**
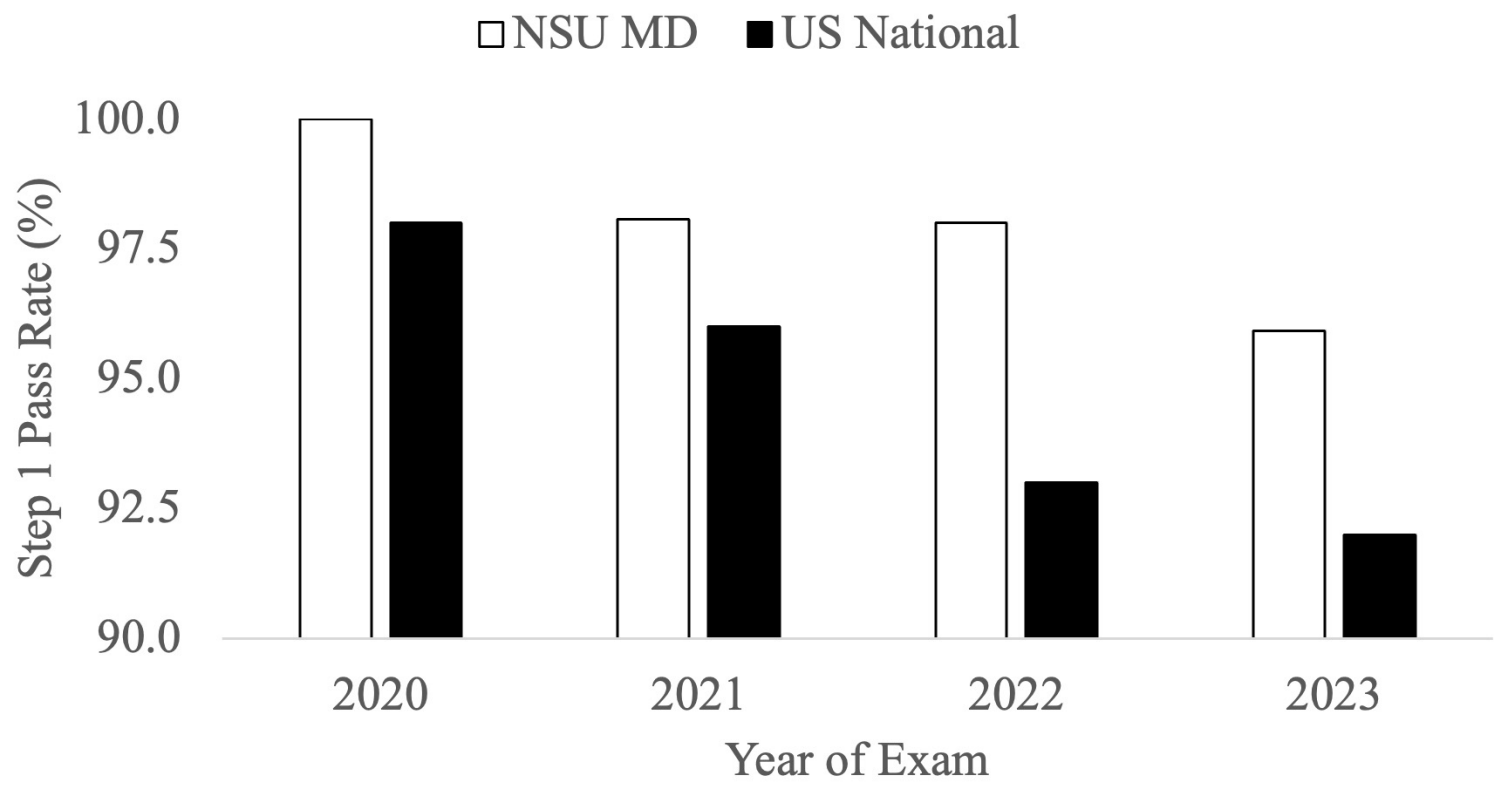
United State Medical Licensing Examination (USMLE) Step 1 first attempt pass rates (%) for NSU MD students (white) compared to national averages (black) for the 2020 to 2023 testing years (USMLE Step 1 nation data is available at: usmle.org/performance-data).

The performance of these four cohorts were compared to the national average Step 1 results for each year. The C/O 2022 cohort who took the USMLE Step 1 in 2020 had a first-time pass rate of 100%, compared to the national average of 98% ( Figure 5). The C/O 2023 cohort who took Step 1 exam in 2021 had an average first-time pass rate of 98%, compared to the national average of 96% ( Figure 5). The two cohorts, C/O of 2024 and 2025, who have taken the USMLE Step 1 as P/F had a pass rate of 98% in 2022 and 96% in 2023, respectively, as compared to the national pass rate available in 2022 of 93%, and 92% in 2023. ( Figure 5).

## Discussion and conclusions

The change of the USMLE Step 1 result reporting from the 3-digit numerical scoring to a binary Pass/Fail (P/F) outcome was a decision met with mixed opinions among the medical education community as well as medical students. Among the stakeholders invited to the Invitational Conference on USMLE Scoring to discuss and propose solutions to the challenges of Step 1 scoring were also medical students who had previously taken Step 1 ( Barone *et al.*, 2019). Until recently, Step 1 has been arguably one of the most important exams a future physician takes during their training. It was initially designed to assist state authorities in granting medical licenses ( Melnick *et al.*, 2002); however, it has evolved far beyond the intended binary indication of competence. The Step 1 scores have become heavily weighted during residency applications to assess a candidate’s strengths and weaknesses ( McGaghie *et al.*, 2011). This directly resulted in low scores becoming a prohibiting factor for students hoping to pursue highly competitive medical specialties ( Chen *et al.*, 2019). A lower-than-average passing score was typically not considered competitive enough for specialties such as dermatology or plastic surgery, etc. ( Gauer & Jackson, 2017). This has resulted in many medical students deciding to change their career trajectory to pursue entirely different specialties, due to poorer Step 1 scores than they believed to make them competitive candidates ( Jones *et al.*, 2021).

The practice exam averages across the four student cohorts examined for this study demonstrate a potentially slower and less rigorous start to Step 1 self-studying during the dedicated preparation block after the change in exam format to P/F. Similarly, self-reported study regiments during the study block showed a decrease in the number of hours per day and number of weeks of dedicated study for the cohorts with P/F Step 1 exam format. This is despite the proposed study plans being similar for all cohorts, as they were uniformly pre-reviewed and approved by the administration (Director of Student Success) ahead of each dedicated study block. The results also indicate students utilized multiple newer study resources as compared to when the examination had a scored format. This change in the student approach and perspective towards Step 1 exam preparation suggests a need to revise the medical school curriculum to better facilitate the student needs in order to be successful on the exam. This may include a shift in the types of resources provided to students throughout their pre-clerkship years that may encompass a variety of resources that have historically not been utilized for Step 1 exam preparation as shown above ( Figure 4). Moreover, incorporation of resources beyond medical knowledge, such as wellness resources and exam taking skills development might be helpful curricular additions.

The goal of the scoring format to change to P/F was to improve the well-being of the first- and second-year medical students by decreasing the stress surrounding the Step 1 exam preparation and performance ( Baniadam *et al.*, 2023). Medical student burnout and depression have been documented in the literature, with Step 1 performance being listed as a contributing factor ( National Academies Press, 2019). It is unsurprising that for most students, the emphasis on one scored exam to determine the upward mobility of medical students' careers was incredibly stressful and potentially led to poorer exam performance as an unintended result ( Mandal *et al.*, 2012; National Academies Press, 2019). When comparing the performance of these cohorts to the national average scores/results ( Figure 5), it may be deduced that irrespective of the format change of the Step 1 exam from a scored to a P/F format, the individual cohorts have continued to perform ~2% above the national average.

Taking this and other factors into account, the rationale for changing the Step 1 exam to the P/F scoring format included relieving some of the stress from first- and second year medical students. A similar rationale has been demonstrated effective, as P/F medical school curriculums have shown to improve mood and group cohesion ( Rohe *et al.*, 2006). This has facilitated an environment of more collaboration amongst peers and less anxiety and toxic competition. Although this may be the intention of the P/F scoring format for Step 1 exams, it is important to evaluate and assess if this change has succeeded in achieving this goal.

Since the most highly demanded specialties will continue to be competitive due to limited spots available, residency directors must look to alternative metrics to assess candidates’ worthiness. One potential unfavorable consequence will likely be that this change will shift the stress on the Step 2 CK, which is currently still graded on a numerical scoring format ( Ehrlich *et al.*, 2021). In effect delaying the stress from Step 1 to Step 2 CK, which provides students with less time to “pivot” to a new specialty if they believe their Step 2 CK score is not competitive enough to be successful in their residency aspirations.

How students divide their time throughout medical school may alter as a result of the USMLE Step 1 going P/F. Students may spend less time preparing for Step 1 and more time on other aspects of their residency applications that will make them a more competitive candidate. These other includes research, extracurricular activities, Step 2 CK preparation, and performance in medical school. This might be because Step 1 may no longer have the same weight on residency applications that it has historically. The students’ three-digit scores on the USMLE Step 1 were one of the main factors in determining competitiveness for residency programs. Instead, it might become more attractive to many students to devote less time to Step 1 preparation and more time to Step 2 CK preparation as it will become the only remaining scored metric for the residence applications. Moreover, as the content assessed on Step 2 CK is significantly more therapeutically relevant than Step 1, this may be viewed as a major victory for medical students as they can allocate more time reinforcing the material that will be more relevant during residency.

Another strategy students may apply to stand out on their applications for residency is by maximizing their research experience. Having more publications and research projects is typically seen as more impressive and is an easy metric for residency programs to use as an initial screening method. Engaging in extracurricular activities such as volunteering in underserved medical clinics, taking on leadership positions in clubs or interest groups, and getting involved in unique opportunities may also serve to show merit. Additionally, accolades such as the Dean’s lists and class rankings, and induction into honor societies such as the Alpha Omega Alpha Society will become more important as they provide an objective method to compare candidate applications.

This might result in residency programs applying a more holistic application review process instead of using score cutoffs. However, since residency programs receive a large number of applications that outnumber the residency spots by many folds, and such a review process will require residency programs to invest more time per application to go over the numerous aspects in detail.

In addition to U.S. MD programs, Doctor of Osteopathic Medicine (DO) students and international medical graduates (IMG) might also be affected by USMLE Step 1 being a P/F exam ( Ehrlich *et al.*, 2021). One of the primary concerns for DOs and IMGs is the increasing difficulty in making their residency program applications more competitive. Strong Step 1 scores in the past have made DOs and IMGs more competitive with MD students for residency spots. However, by having a competitive Step 2 score, their research experience, extracurricular activities, and letters of recommendation, DOs and IMGs may still be able to have a competitive application. Strong letters of recommendation in particular have been and will continue to be crucial, especially for DOs and IMGs.

Overall, the primary objective to transition the scored Step 1 exam to a P/F format was to reduce the stress it may lead to for the medical students. However, the residency programs and specialties are not becoming more or less competitive from this change, and the stress that accompanies the competition and uncertainty is still prevalent. It is only by adopting an internal locus of control that students can make themselves competitive applicants.

While a potential limitation of this study’s findings may be the relatively small sample size of four cohorts from only one medical school, the themes illustrated by these data may be representative of a more global shift in student mindsets and practices going forward. For further analysis and as a future direction of this study, the student performance, perceptions and approach towards Step1 exam preparation from other medical schools can be collected and compared. This will help build a larger data set and a collaborative effort that can be adopted by the medical schools as potential approaches to support and facilitate medical students during their standardized licensing examinations.

Future directions that medical schools can employ to facilitate training their students to be better able to cope with the stress and uncertainty and competitiveness of the USMLE board certification by incorporating tools and educational objectives directed towards having a learning/growth mindset in their curriculums. Moreover, providing more wellness resources and career counselling to the students throughout their medical school training will ensure better equipping the students for their future. Other resources may include providing newer resources for the Step 1 exams catered towards a P/F format. Furthermore, incorporating dedicated time in the curriculum for activities such as research, community service, and leadership opportunities will allow medical school students to diversify their skillset and make their residency program applications more competitive. Future studies may wish to collect paired information from individual students, on preparation and outcomes, to better understand trends in study methods and outcomes, in order to better advise future cohorts.

In conclusion, it is imperative to emphasize that a medical student’s success as a board-certified physician depends heavily on the training provided to them at their medical schools. Now, more than ever, it is important to include training students to be on skills that equip them to be adaptable, practice foresight, and prioritize wellness in their approach.

## Data Availability

Harvard Dataverse: Step 1 Preparation Student Evaluation Questionnaire. https://doi.org/10.7910/DVN/LOT6TD ( Carnevale, 2024) Harvard Dataverse: Step 1 Prep Data Prasad Perez Carnevale. https://doi.org/10.7910/DVN/IXIUMB ( Carnevale, 2023). This dataset contains the cohort data, no data from individual student study plans were directly analyzed by the authors. Data are available under the terms of the Creative Commons Zero "No rights reserved" data waiver (CC0 1.0 Public domain dedication).
