# Supplementary material for: Comparison of Medical students' preparation for the United States Medical Licensing Examination (USMLE) Step 1 before and after transition to Pass/Fail
Source: MedEdPublish (2016). 2025 Jul 28;14:20. Originally published 2024 Apr 12. [Version 3] doi: 10.12688/mep.19975.3 (PMC12322661; doi:10.12688/mep.19975.3)
Supplement: Peer review history

# Reviewer report: Reviewer response for version 3

**DOI:** 10.21956/mep.22696.r42570

**Published:** 2025-08-04

**Authors:** Houman Goudarzi

**Affiliations:** 1 Hokkaido University, Sapporo, Japan

I believe the authors have made substantial improvements, and the manuscript has been strengthened. Therefore, I am pleased to approve it.

Have any limitations of the research been acknowledged?

Partly

Is the work clearly and accurately presented and does it cite the current literature?

Partly

If applicable, is the statistical analysis and its interpretation appropriate?

Partly

Are all the source data underlying the results available to ensure full reproducibility?

Yes

Is the study design appropriate and is the work technically sound?

Partly

Are the conclusions drawn adequately supported by the results?

Partly

Are sufficient details of methods and analysis provided to allow replication by others?

Partly

Reviewer Expertise:

NA

I confirm that I have read this submission and believe that I have an appropriate level of expertise to confirm that it is of an acceptable scientific standard.

# Reviewer report: Reviewer response for version 2

**DOI:** 10.21956/mep.21954.r38277

**Published:** 2024-09-09

**Authors:** Houman Goudarzi

**Affiliations:** 1 Hokkaido University, Sapporo, Japan

The authors of the current manuscript evaluated the impact of the transition from three-digit scores to Pass/Fail (P/F) grading in the USMLE Step 1 on average practice exam scores, self-reported study duration, study intensity, and study resources used by medical students at a US medical school. This topic is important, and such an assessment is both valuable and novel, given the limited number of studies focused on this area. However, the study has several limitations.

**Below, I outline my comments, which I hope will help the authors improve the manuscript:**

**Major Comments:**

**1. Participant Characteristics:**

 Information on the characteristics of the participants is not presented. The authors used data from four cohorts of second-year medical students, with a total sample size of 204. However, there is no information regarding the number of participants in each cohort, sex ratio, age, etc. It is important to provide this basic demographic data, as these characteristics could influence the outcomes being studied. Additionally, the selection criteria for this study are unclear. Were all students in a class included in each cohort, or was a subpopulation selected? When and how was the study explained to the students? Were students able to opt out? What was the response rate? This information is crucial, as the methodology dire.

**2. Statistical Analysis:**

 There are several areas where the statistical analysis could be improved:

 a) Figure 1 interpretation: The authors use range bars to represent the highest and lowest scores for each cohort rather than standard deviations. They state that “ No statistically significant difference was observed in cohort performance averages before and after the transition to P/F. However, it can be seen that the distributions between high and low scores were wider for the cohorts preparing for the P/F Step 1 than for cohorts who prepared for the scored Step 1 exam”. However, outliers can significantly widen score ranges, and this alone does not justify a conclusion of difference between the cohorts. Additionally, CBSE 2 in 2021 (with scored USMLE Step 1) shows a wider range than the P/F cohorts of 2022 and 2023, which contradicts the authors' interpretation. I believe this part of the analysis is misinterpreted, and I recommend the authors consult a biostatistician to conduct additional analyses to ensure scientific accuracy in interpreting these results.

 b) Figures 2 and 3: These figures display self-reported study duration and study intensity. I suggest combining them to show total study hours for each cohort, as this would facilitate easier comparisons. Furthermore, p-values should be provided to indicate significant differences between the cohorts, rather than relying on vague comparisons and interpretations. A combined figure may offer clearer insights into differences in study behaviors across the cohorts.

 c) Cohort Differences: Significant differences between the 2020 and 2021 cohorts (scored USMLE Step 1), as well as between the 2022 and 2023 cohorts (P/F USMLE Step 1), are visible in Figures 2, 3, and 4. However, these differences are not discussed in the manuscript. Could these discrepancies be due to variations in cohort characteristics or external factors like the COVID-19 pandemic? It would be helpful for the authors to address these differences in the Discussion section as a potential limitation.

 d) Sample Size Calculation: Did the authors perform a sample size calculation to determine the necessary number of participants for adequate statistical power? Given the likely sample size of approximately 50 participants per cohort, it would be beneficial to clarify whether this number is sufficient to provide robust statistical comparisons across the cohorts.

**3. De-identified Data:**

 As the authors note, "Since the data obtained was deidentified, there was no way to correlate individual student effort and exam performance outcomes", this is a significant limitation, as it restricts the possibility of conducting further analysis to explore additional correlations.

**Minor Comments:**

**1. Figures 1-5:**

 Adding a Y-axis bar to the figures would improve readability and help readers better see and interpret the results.

**2. Figure 4:**

 The authors present data as raw student numbers rather than percentages. If the cohorts vary in size, it would be more appropriate to present the data as percentages to allow for a more accurate comparison.

 Taken together, the authors have made minor revisions; further substantial improvements are needed to enhance the manuscript's scientific rigor. A neutral interpretation of the data, avoiding speculation, and presenting results supported by robust statistical analysis will significantly improve the clarity and impact of the study.

Have any limitations of the research been acknowledged?

Partly

Is the work clearly and accurately presented and does it cite the current literature?

Partly

If applicable, is the statistical analysis and its interpretation appropriate?

Partly

Are all the source data underlying the results available to ensure full reproducibility?

Yes

Is the study design appropriate and is the work technically sound?

Partly

Are the conclusions drawn adequately supported by the results?

Partly

Are sufficient details of methods and analysis provided to allow replication by others?

Partly

Reviewer Expertise:

Medical education, internal medicine, epidemiology

I confirm that I have read this submission and believe that I have an appropriate level of expertise to confirm that it is of an acceptable scientific standard, however I have significant reservations, as outlined above.

# Reviewer report: Reviewer response for version 2

**DOI:** 10.21956/mep.21954.r37823

**Published:** 2024-09-04

**Authors:** Rehan Ahmed Khan, Dr. Mashaal Sabqat Sabqat

**Affiliations:** 1 Riphah International University, Islamabad, Islamabad Capital Territory, Pakistan

The authors have updated Figure 5 and have addressed some of the comments on the first version of the article. However, a few aspects still need elaboration/correction.

 The title does not align with the Methods and Results section. There is no mention of any student perspectives in the article. The study design and sampling method are not explicitly mentioned anywhere in the article. Was informed consent obtained from the students? Was voluntary participation ensured? The aim of the study in the Introduction section is ‘to evaluate students’ perceptions of their efforts in Step 1 preparation, as compared to cohort performances…’. However no such comparative analysis, like between number of study hours or resources used with student test performance is given in the Results. The discussion section is clearer now with revisions. The conclusion however needs more clarity. The last lines of the section ‘In conclusion, it is imperative to emphasize that a medical student’s success as a board-certified physician depends heavily on the training provided to them at their medical schools. Now, more than ever, it is important to include training students to be on skills that equip them to be adaptable, practice foresight, and prioritize wellness in their approach’ are not aligned with the aim of the study.

Have any limitations of the research been acknowledged?

Partly

Is the work clearly and accurately presented and does it cite the current literature?

Yes

If applicable, is the statistical analysis and its interpretation appropriate?

Partly

Are all the source data underlying the results available to ensure full reproducibility?

Partly

Is the study design appropriate and is the work technically sound?

Partly

Are the conclusions drawn adequately supported by the results?

Partly

Are sufficient details of methods and analysis provided to allow replication by others?

Partly

Reviewer Expertise:

Medical Education, Surgery

We confirm that we have read this submission and believe that we have an appropriate level of expertise to confirm that it is of an acceptable scientific standard, however we have significant reservations, as outlined above.

# Reviewer report: Reviewer response for version 2

**DOI:** 10.21956/mep.21954.r37782

**Published:** 2024-08-28

**Authors:** Tracey Weiler

**Affiliations:** 1 Florida International University, Miami, Florida, USA

Dear Authors, 

 Thank you for the revisions to the manuscript.  There are a number of comments from my previous review that have not yet been addressed.  I recommend revising again based on some of those comments, in addition to the ones below.

**Title:**

- I don't think that the paper presents medical student perspectives on the Step 1 transition to P/F.  This paper is about differences in study time and study behavior before or after P/F.  Change the title to better reflect the content of the paper. 

Throughout the paper, please change the word regiment to regimen

**Introduction:**

- At the end of the 1st paragraph, please reference the USMLE Step Exam Website. 

**Methods Data Collection:**

- The first sentence is confusing.  ... cohort practice test data were analyzed for comparative analysis...  Please reword for clarity. 
- Given that the results of 2025 CBSE and CBSSA were reported as weighted averages, it would be helpful to know how they were translated for the purposes of comparison. 
- Figure 1 legend.  Improve text for clarity and remove duplicated information related to range bars. 
- Figure 2/3.  Weeks of studying without hours of studying does not provide enough information.  Someone that studies for 8 weeks for 6 hours a day, is different than someone else who studies for 6 weeks at 12 hrs a day.  It would be more helpful to merge these two graphs into one. If possible convert this graph to hours per week, or hours total to standardize. 

**Discussion:**

- Discussion Paragraph 3
  When Step 1 was scored, students had 2-3 major exams before their residency match.  Now that CS has been removed, and Step 1 is P/F, only the Step 2 CK is left as a scored exam prior to residency.  It seems as if the current situation is more stressful than the previous 2 exam situation! 
- Discussion Paragraph 5. 
  The sentence starting "In effect delaying..." is a fragment.  
- Discussion Paragraph 7
  Increasing research and other extracurriculars takes time away from academics, and that in itself is a stressor that can impact student wellness. 
- Discussion Paragraph 10
  This paragraph is confusing .  Rewrite to improve clarity or remove entirely? 

Have any limitations of the research been acknowledged?

Partly

Is the work clearly and accurately presented and does it cite the current literature?

Yes

If applicable, is the statistical analysis and its interpretation appropriate?

No

Are all the source data underlying the results available to ensure full reproducibility?

Yes

Is the study design appropriate and is the work technically sound?

Partly

Are the conclusions drawn adequately supported by the results?

Partly

Are sufficient details of methods and analysis provided to allow replication by others?

Partly

Reviewer Expertise:

Medical education, Genetics education

I confirm that I have read this submission and believe that I have an appropriate level of expertise to confirm that it is of an acceptable scientific standard, however I have significant reservations, as outlined above.

# Reviewer report: Reviewer response for version 2

**DOI:** 10.21956/mep.21954.r37781

**Published:** 2024-08-12

**Authors:** Adnan Innab

**Affiliations:** 1 King Saud University, Riyadh, Saudi Arabia

Dear Authors,

 Thanks for giving me the chance again to review this study again. I hope my comments are helpful for you.

**Some of my previous comments were not addressed:**

- Please address the research design (i.e., cross-sectional, correlational, descriptive design).
- Please address the inclusion and exclusion criteria.
- Please put a heading of “Implications and Recommendations” before the following paragraph ''Future directions that medical schools can employ to facilitate training their students to be better able to cope with the stress and uncertainty and competitiveness of the USMLE board certification by incorporating tools and educational objectives directed towards having a learning/growth mindset in their curriculums…..”

Have any limitations of the research been acknowledged?

Yes

Is the work clearly and accurately presented and does it cite the current literature?

Partly

If applicable, is the statistical analysis and its interpretation appropriate?

Yes

Are all the source data underlying the results available to ensure full reproducibility?

Yes

Is the study design appropriate and is the work technically sound?

No

Are the conclusions drawn adequately supported by the results?

Partly

Are sufficient details of methods and analysis provided to allow replication by others?

Yes

Reviewer Expertise:

Statistics, Methodology, Nursing Education

I confirm that I have read this submission and believe that I have an appropriate level of expertise to confirm that it is of an acceptable scientific standard, however I have significant reservations, as outlined above.

# Reviewer report: Reviewer response for version 2

**DOI:** 10.21956/mep.21954.r38078

**Published:** 2024-08-09

**Authors:** Eileen Hennrikus

**Affiliations:** 1 Penn State College of Medicine, Hershey, PA, USA

**Abstract:**

 Recommend that you clarify that of your 4 year cohort, 2 years took scored Step 1 and 2 years took P/F Step 1.

**Introduction:**

*These changes may more drastically reshape the nascent academic cultures of new medical schools whose student bodies lack generations of vetted alumni with track records beyond residency that can inspire confidence and promote stability of study practices within current medical school undergraduates.* 

- Do you really mean that established medical schools have a reputation (eg. Harvard) that benefits student applications when scores go to P/F?
- CBSE taken before study block
- CBSSA and UWorld taken during study block

**Results:**

*No statistically significant difference was observed in cohort performance averages before and after the transition to P/F.* *However, it can be seen that the distributions between high and low scores were wider for the cohorts preparing for the P/F Step 1 (black and pattern fill bars, Figure 1* *)  *

- This looks to be true only for UWorld. Was this statistically significant?

 
*There was no way to correlate individual student effort and exam performance outcomes, but it may be inferred that more weeks of studying would likely lead to a higher level of preparedness, compared to fewer weeks of studying.*

- This is not necessarily true.  More than 8 weeks means that the students are trying to get their practice scores as high as possible.  7-8 weeks may be sufficient to pass.

*The goal of the scoring format to change to P/F was to improve the well-being of the first- and second-year medical students by decreasing the stress surrounding the Step 1 exam preparation and performance*

- So do you think that Studying 6-10 hours a day rather than 10-12 hours a day shows less stress and better well-being?
- Study resources may have changed to UWorld with time as students narrowed down their choices, choosing those resources that provide better bang for the buck for passing step 1.

*This change in the student approach and perspective towards Step 1 exam preparation suggests a need to revise the medical school curriculum to better facilitate the student needs in order to be successful on the exam. This may include a shift in the types of resources provided to students throughout their pre-clerkship years that may encompass a variety of resources that have historically not been utilized for Step 1 exam preparation as shown above*

- I counted 24 different study resources that you listed.  How about saying something about the ‘hidden curriculum’ that students don’t feel that the 2 years of classes have prepared them for Step 1 and they have to go to outside resources. How about medical schools provide the instruction and resources that will help students pass step 1, so that they don’t have to depend so heavily on outside, online resources.
- Is the National pass rate change statistically significant.
- Going from 96/98% down to 93/92% seems significant.

*It is unsurprising that for most students, the emphasis on one scored exam to determine the upward mobility of medical students' careers was incredibly stressful and potentially led to poorer exam performance as an unintended result ( Mandal et al., 2012* *; National Academies Press, 2019* *).*

- Now it really is only one exam: Step 2.  Isn’t the stress simply displaced from second year  (Step 1) to third year (Step 2)?
- To assess students, isn’t better to have many points on the plot rather than one?  Wouldn’t it be better to go back to multiple grades through medical school, rather than just one?

*When comparing the performance of these cohorts to the national average scores/results ( Figure 5* *), it may be deduced that irrespective of the format change of the Step 1 exam from a scored to a P/F format, the individual cohorts have continued to perform ~2% above the national average.*

- Perhaps your med school teaches more appropriately to what is on Step 1, vs other schools.
- Do you have grades in medical school? Preclinical? Clinical?

**Discussion & Conclusion:**

*A similar rationale has been demonstrated effective, as P/F medical school curriculums have shown to improve mood and group cohesion ( Rohe et al., 2006* *). This has facilitated an environment of more collaboration amongst peers and less anxiety and toxic competition. Although this may be the intention of the P/F scoring format for Step 1 exams, it is important to evaluate and assess if this change has succeeded in achieving this goal.*

- Absolutely.  Change needs assessment of outcomes.

* In effect delaying the stress from Step 1 to Step 2 CK, which provides students with less time to “pivot” to a new specialty if they believe their Step 2 CK score is not competitive enough to be successful in their residency aspirations.*

- They’re taking step2 earlier, with less clinical experience and with little time to adjust their specialty desires and applications.

*How students divide their time throughout medical school may alter as a result of the USMLE Step 1 going P/F. Students may spend less time preparing for Step 1 and more time on other aspects of their residency applications that will make them a more competitive candidate. These other includes research, extracurricular activities, Step 2 CK preparation, and **performance in medical school.***

- Perhaps less time studying question banks and more time on real life learning. Medical schools need to make real life learning available.

*Additionally, accolades such as the Dean’s lists and class rankings, and induction into honor societies such as the Alpha Omega Alpha Society will become more important as they provide an objective method to compare candidate applications.*

- Without class grades how are these decided?

*Overall, the primary objective to transition the scored Step 1 exam to a P/F format was to reduce the stress it may lead to for the medical students. However, the residency programs and specialties are not becoming more or less competitive from this change, and the stress that accompanies the competition and uncertainty is still prevalent. It is only by adopting an internal locus of control that students can make themselves competitive applicants*.

- Agree. You can’t homogenize all the students and then throw them into a highly competitive situation. That will only increase stress.

*Future studies may wish to collect paired information from individual students, on preparation and outcomes, to better understand trends in study methods and outcomes, in order to better advise future cohorts.*

- Actually, why don’t you look at med schools who ‘teach to the test’ and med schools who don’t. If the makers of the test believe that step1 is core information that students should pass before moving on to clinical rotations, what wrong with teaching that information along with test taking skills?

*In conclusion, it is imperative to emphasize that a medical student’s success as a board-certified physician depends heavily on the training provided to them at their medical schools.*

- I don’t think you proved this in your paper.  You showed the use of an abundant number of ‘outside med school’ resources that students go to, to supplement their classes.

*Now, more than ever, it is important to include training students to be on skills that equip them to be adaptable, practice foresight, and prioritize wellness in their approach.*

- This is hyperbole, omit. This has not been supported by the study and is opinion based.

Have any limitations of the research been acknowledged?

Yes

Is the work clearly and accurately presented and does it cite the current literature?

Yes

If applicable, is the statistical analysis and its interpretation appropriate?

Partly

Are all the source data underlying the results available to ensure full reproducibility?

Yes

Is the study design appropriate and is the work technically sound?

Yes

Are the conclusions drawn adequately supported by the results?

Partly

Are sufficient details of methods and analysis provided to allow replication by others?

Yes

Reviewer Expertise:

Medical Education

I confirm that I have read this submission and believe that I have an appropriate level of expertise to confirm that it is of an acceptable scientific standard, however I have significant reservations, as outlined above.

# Reviewer report: Reviewer response for version 1

**DOI:** 10.21956/mep.21392.r37067

**Published:** 2024-09-04

**Authors:** Vivek Bhat, Dhwani Ravi

**Affiliations:** 1 St John's Medical College, Bangalore, Karnataka, India; 2 St John’s Medical College, Bangalore, Karnataka, India

The authors present a single center study assessing medical students' perspective regarding the USMLE Step 1 transition from pass/ fail to scored. 

**General comments:**

 1. The authors have not provided sufficient background, and have missed seminal literature on the impact of the USMLE Step 1 transition.

 2. Solely based on the current writing, I have significant concerns about how the study was conducted, and how much choice the students had.

 3. The phrasing could be improved in several places - for example 'The transition of the United States Medical Licensing Exam: Step 1 to Pass/Fail (P/F), from scored,..'; - this could say '..Step 1 from a scored exam to a pass/ fail..'

 '....medical licenses; however, it has evolved...' - this should say 'however, it evolved...'

 4. There are numerous passages that are improperly placed or confusingly phrased - for example, the beginning of the methods, and the end of the introduction. I recommend the authors take a second look at their manuscript to improve flow. Sentences may be divided into smaller sentences to improve readability.

 5. The results fail to mention even the underlying numbers, for example, sample size.

**Section-wise comments:**

**Abstract:**

 1. The aim of the study needs to be mentioned.

 2. The methods should make it clear that some students had taken a P/F exam, while others had taken a scored exam.

 3. The results need to have specific details - the authors say 'median number of hours/ day' but have not provided the actual number.

 4. The conclusion is excessively broad. Read in isolation, it is vague and provides no insight. Preparedness for what? Resources and support for what? What are the potential implications of the authors' findings?

**Introduction:**

 1. 'While this decision was mostly celebrated...' - such a statement is controversial and needs robust referencing if the authors intend to retain it. 

 2. Further, the authors did not adequately summarize the potential impact of the Step 1 transition. The sentences are confusingly phrased. For example, I fail to see how a strong alumni base is related to current study practices in a medical school. If the authors had said something like 'the loss of an objective metric in Step 1 may lead to increased focus on other relatively ambiguous metrics like school prestige, diminishing residency prospects for students from lower-ranked schools', I would understand. 

 Please take a look at Reference 1.

**Methods:**

 1. It does not appear that students had a choice in participation - consent was waived. How did this meet the criteria for IRB exemption given the apparent lack of privacy in these students' dedicated periods? What is the justification for students, well into their 20s, needing to run their study plans by administration?

 2. Which specific NBME examinations were administered? 

**Results:**

 1. Some basic details have not been presented - how many students were in each class? How many had complete data? In fact, most of the section fails to mention the underlying numbers.

 2. All the graphs should have the underlying numbers presented. It is generally recommended to use tables and avoid graphs unless absolutely necessary.

 3. The authors should present inferential statistics to highlight significant differences.

**Discussion & Conclusion:**

 1. The very first sentence contradicts an earlier statement where the transition was apparently celebrated.

 2. The authors also did not mention that not only is the stress shifted to Step 2, there is no opportunity to demonstrate score improvement, as this is now the only scored exam.

 3. The use of newer resources could merely indicate that newer resources are available, rather than a need to revise medical school curriculum. Medical school curriculum has long been incongruent with the USMLE, and the presence of newer resources is not exactly related.

 4. There are significantly more limitations to this study - one being that it relied on self reports, which may have not been accurate.

 5. The conclusions are not connected to the results/ discussion at all. For the authors to talk about success as a board-certified physician, they need to discuss literature connecting USMLE scores to clinical outcomes, in-training exams, board certification rates, etc.

Have any limitations of the research been acknowledged?

No

Is the work clearly and accurately presented and does it cite the current literature?

No

If applicable, is the statistical analysis and its interpretation appropriate?

Partly

Are all the source data underlying the results available to ensure full reproducibility?

Yes

Is the study design appropriate and is the work technically sound?

No

Are the conclusions drawn adequately supported by the results?

No

Are sufficient details of methods and analysis provided to allow replication by others?

No

Reviewer Expertise:

Internal medicine, cardiology, medical education

We confirm that we have read this submission and believe that we have an appropriate level of expertise to state that we do not consider it to be of an acceptable scientific standard, for reasons outlined above.

## References

[1] : The US Residency Selection Process After the United States Medical Licensing Examination Step 1 Pass/Fail Change: Overview for Applicants and Educators. *JMIR Med Educ* .2023;9: 10.2196/37069 e37069 10.2196/37069 36607718 PMC9862334

[2] : Objective Measures Needed — Program Directors’ Perspectives on a Pass/Fail USMLE Step 1. *New England Journal of Medicine* .2020;382(25) : 10.1056/NEJMp2006148 2389-2392 10.1056/NEJMp2006148 32558467

# Reviewer report: Reviewer response for version 1

**DOI:** 10.21956/mep.21392.r36634

**Published:** 2024-06-04

**Authors:** Adnan Innab

**Affiliations:** 1 King Saud University, Riyadh, Saudi Arabia

The authors need provide further details about the study. Most of my comments, if not all, were not addressed. 

 Please address the aim of the study in the abstract. The research design and settings must be also addressed in the abstract section.   Please revise the keywords according to MeSH   Where is the purpose of the study ? The specific aims OR research questions were not addressed.   Methods: The research design must be clear. Is it a quasi- experimental study.  What was the sampling method ?   Human Subjects: It is not enough to state that this study recived IRB approval. You must indicate how the rights for human subjects were maintained (i.e., privacy, confidentiality, anonymity, voluntarily participation…etc).       The results and discussion are impressing. The future direction for research and practice was highlighted. The conclusion needs further details.

Have any limitations of the research been acknowledged?

Yes

Is the work clearly and accurately presented and does it cite the current literature?

Partly

If applicable, is the statistical analysis and its interpretation appropriate?

Yes

Are all the source data underlying the results available to ensure full reproducibility?

Yes

Is the study design appropriate and is the work technically sound?

No

Are the conclusions drawn adequately supported by the results?

Partly

Are sufficient details of methods and analysis provided to allow replication by others?

Yes

Reviewer Expertise:

Statistics, Methodology.

I confirm that I have read this submission and believe that I have an appropriate level of expertise to state that I do not consider it to be of an acceptable scientific standard, for reasons outlined above.

# Reviewer report: Reviewer response for version 1

**DOI:** 10.21956/mep.21392.r36638

**Published:** 2024-05-14

**Authors:** Tracey Weiler

**Affiliations:** 1 Florida International University, Miami, Florida, USA

The manuscript entitled "Medical Students' perspective on the United States Medical Licensing Examination (USMLE) Step 1 transition to Pass/Fail" investigates the difference in student performance on standardized practice examinations before and after the Step 1 exam moved to P/F.  This is an important topic.  We need to know how students are treating the exam, and whether their behaviours are setting them up for success.  A major justification for shifting to P/F was to decrease stress and increase well being.  It is important to know if the change was successful in achieving its goal, while maintaining competency.  The strengths of this paper are in the standardization of the dedicated study period with standardized tests given at approximately the same time for individual students.  A significant weakness of the paper is in the statistical analysis.  To increase power, the scored and P/F cohorts could have been combined.  The significance between the two groups should have been reported throughout.  Furthermore, although wellbeing/stress is a significant driver of the move to P/F, it was not a part of the study, but rather only part of the conclusions. 

 In the Title...  I don't think that "Medical student's perspective" is appropriate here.  This paper did not talk about the perspective of the med students. 

 In the abstract methods and results, the word should be regimens, not regiments.  There is mention of well-being in the abstract, but there is no mention in the Introduction, Methods, Results.  Was there any assessment of wellbeing in the surveys at all?  If not, this should be removed.  If yes, then it needs to be incorporated into the intro, methods, and results as well. 

 In the Introduction, Step 3 is taken after the completion of MD or DO degree, and typically near the end of the 1st year of GME residency training. https://www.usmle.org/bulletin-information/eligibility

 At the end of the introduction... I would argue that the transition to P/F has also harmed the graduates of new medical schools.  When the Step1 score is no longer an option, and there is no significant track record of success beyond residency for students from new medical schools, prestigious and highly-regarded programs will then choose residents from older medical schools with a track record of success. 

 In the methods | Sample participants...  Did each cohort have exactly the same MCAT and GPA?  That is very surprising. How many students in each of the classes?

 In the methods | Data collection...  the first sentence is poorly worded.  "... test data were analyzed for comparative analysis to determine if there were any trends and correlations...".  Also - I would like to see the wording of the questions for the survey. 

 In the methods | Data collection...  It would be helpful to have a better idea of how the exams are scheduled (or not) with respect to each other. This information is in the first part of the results section. Should be moved to methods. 

 In the methods | Data collection... Were there any minimum scores on these exams required/recommended prior to taking Step1?   What were the "pass" scores for each of the four years?  I think they were increased at one point during this time frame. 

 In the methods | Data collection...  it would be helpful to know how the 2025 CBSE and CBSSA exam scores were manipulated to put them in the same graph as exams with direct percentages. 

 In the methods | Data analysis...  statistical analyses should have been performed.  Correlations between time and performance on exams could have been determined. 

 In the Results | 1st paragraph.  Timing of exams should be in methods. What are the demographics of the class?  M/F?  Age?  Race/ethnicity? 

 In the Results | end of 1st paragraph...  I don't understand what is meant by the statement "differences can only be inferred". 

 In the Results | Figure 1...  If the "error bars" represent the high and low scores, they are not error bars.  Is there any significant difference between the scored and P/F for any of the exams?  If not, then why not average scored and P/F to get a larger N, and then check to see if there is a significant difference in performance between the two for any of the exams. 

 In the Results | 2nd Paragraph...  If you have both weeks and hours per day, can you calculate total hours of studying?  This would be a more standard measure than simple weeks.  4h/day for 4 weeks, and 8h/day for 4 weeks are very different things. 

 In the Results | Figures 2 & 3...  can these two figures be merged into one that is total hours studied?  Then, test for significant differences between scored and P/F.  Could also merge the two scored vs. P/F if there are no significant differences between the two years. 

 In the Results | Figure 4...  were there any differences in the medical education that may have resulted in the changes in the resources used by the students?  ie.  Was Sketchy a thing in 2021 b/c the instructor or the tutors recommended it as prep/revision work? 

 In the Results | End of Paragraph 4 and Figure 4...  It would be helpful to have a better description of these resources, including URLs where appropriate.  What is "notes"?  Is it the in-class notes the student took?  What is Costanzo?  The bible of physiology?  or the BRS for Physiology?  or? What is "High Yield"?  

 In the Results | Table 1and Paragraph 5...   I don't understand this table.  I find it hard to believe that only 26% of test takers used UWorld.  I would expect that all test takers used UWorld (especially since they were mandated practice tests).  How was this data collected and analyzed? Was the question a multiple answer question?  The order of importance seems correct, the numbers seem off.   Once these numbers are fixed, it would be helpful to know the difference between the scored and P/F.  Put a column with the delta between the two?  Is there any significant difference here?   Also - is the diversity of resources in the P/F perhaps due to the availablility of new resources more recently?  

 In the Results | Paragraph 6...  this analysis does not situate Step 1 exam performance with any of the previous data.  Can you make correlations between performance on the practice exams and on the scored exams?  Step 1 pass rate is not a great metric here. 

 In the Discussion and Conclusions | Paragraph 2...  The claims about the slower and less rigorous start to studying during dedicated cannot really be made without some statistical analysis of the differences.  CBSE exams are before dedicated even begins.  The worst CBSSA mean score is in the scored cohort 2020.  If you are going to make a claim like this, you probably need to create a slope of exam performance over time to see the differences, and then calculate significance. 

 Can the analyses identify one of these practice exams that can predict performance on Step 1? Could you use one of the practice exam scores as a competency proxy for actual performance on the Step?

 As per previous comment about study resources, newer resources become available, and students use them.  If they weren't available in 2020, then it isn't something startling.  Perhaps the comparison should be amongst all resources that were available to all four cohorts, and then the others can be clumped together.  Given that there is no mention of wellness resources and exam taking skills to this point, I do not see how these conclusions can be made. 

 In the Discussion and Conclusions | Paragraphs 3 & 4...  There is no mention of wellbeing throughout the rest of the paper.  Do you have any data that supports the change to P/F has improved wellbeing, mood, group cohesion in your classes? 

 In the Discussion and Conclusions | Paragraphs 5,  6 & 7...  I think these paragraphs should include a discussion of the consequences to medical student competency if they are focusing on research, or extracurricular activities, or Step 2, etc.  What is the outcome of the actions these students are taking? 

 In the Discussion and Conclusions | Paragraphs 5 & 8...  The last sentences, "In effect..."  & "However, since residency programs..." are sentence fragments. 

 In the Discussion and Conclusions | Paragraph 9...  I don't see the relevance of this.

 In the Discussion and Conclusions | Paragraph 10... I think it is relevant that the transition to P/F has shifted all stress to one scored exam (CK),  when it used to be distributed between two exams and the CS exam as well.

 A limitation of the study is the aggregate nature of the data, and the inability to correlate specific study plans for an individual with specific student performance.   

 Future work would include measuring stress and wellbeing before, during the course of the Step 1 and Step 2 dedicated period to see how it is changing over the course of the med student's training. 

 The conclusions related to extra support, training coping strategies, etc.  are not supported by the rest of the paper.  These should be woven throughout or eliminated.

Have any limitations of the research been acknowledged?

Partly

Is the work clearly and accurately presented and does it cite the current literature?

Yes

If applicable, is the statistical analysis and its interpretation appropriate?

No

Are all the source data underlying the results available to ensure full reproducibility?

Yes

Is the study design appropriate and is the work technically sound?

Partly

Are the conclusions drawn adequately supported by the results?

Partly

Are sufficient details of methods and analysis provided to allow replication by others?

Partly

Reviewer Expertise:

Medical education, Genetics education

I confirm that I have read this submission and believe that I have an appropriate level of expertise to state that I do not consider it to be of an acceptable scientific standard, for reasons outlined above.
